# An endangered flightless grasshopper with strong genetic structure maintains population genetic variation despite extensive habitat loss

**DOI:** 10.1002/ece3.7428

**Published:** 2021-04-04

**Authors:** Ary A. Hoffmann, Vanessa L. White, Moshe Jasper, Hiromi Yagui, Steve J. Sinclair, Michael R. Kearney

**Affiliations:** ^1^ Pest and Environmental Adaptation Research Group Bio21 Institute School of BioSciences The University of Melbourne Parkville Victoria Australia; ^2^ School of BioSciences The University of Melbourne Melbourne Victoria Australia; ^3^ Department of Environment, Land, Water and Planning Arthur Rylah Institute Heidelberg Victoria Australia

**Keywords:** fragmentation, grassland, isolation by distance, *Keyacris*, morabine, small population area

## Abstract

Conservation research is dominated by vertebrate examples but the shorter generation times and high local population sizes of invertebrates may lead to very different management strategies, particularly for species with low movement rates. Here we investigate the genetic structure of an endangered flightless grasshopper, *Keyacris scurra*, which was used in classical evolutionary studies in the 1960s. It had a wide distribution across New South Wales (NSW) and Victoria in pre‐European times but has now become threatened because of land clearing for agriculture and other activities. We revisited remnant sites of *K. scurra*, with populations now restricted to only one area in Victoria and a few small patches in NSW and the Australian Capital Territory (ACT). Using DArtseq to generate SNP markers as well as mtDNA sequence data, we show that the remaining Victorian populations in an isolated valley are genetically distinct from the NSW populations and that all populations tend to be genetically unique, with large *F*_ST_ values up to 0.8 being detected for the SNP datasets. We also find that, with one notable exception, the NSW/ACT populations separate genetically into previously described chromosomal races (2*n* = 15 vs. 2*n* = 17). Isolation by distance was detected across both the SNP and mtDNA datasets, and there was substantial differentiation within chromosomal races. Genetic diversity as measured by heterozygosity was not correlated with the size of remaining habitat where the populations were found, with high variation present in some remnant cemetery sites. However, inbreeding correlated negatively with estimated habitat size at 25–500 m patch radius. These findings emphasize the importance of small habitat areas in conserving genetic variation in such species with low mobility, and they highlight populations suitable for future translocation efforts.

## Graphical abstract

*Keyacris scurra* was an abundant invertebrate in southeastern Australia whose distribution has declined drastically leading to its current threatened status. Here we use SNP markers to show that the populations are highly structured across its remnant range but also that remnant populations contain high levels of genetic variation. Inbreeding levels, but not genetic variation, appear to be correlated with the size of habitat patches.

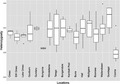

## 1 INTRODUCTION

As with other animals, terrestrial invertebrates are increasingly being threatened by habitat destruction, climate change, invasive species, pesticides, and other threats connected to human activities (Black & Vaughan, 2009; Hafernik, 1992; Wagner & Van Driesche, 2010). Thus, terrestrial invertebrate population declines and extinction rates over the last few 100 years can match those of vertebrates and vascular plants (Harvey et al., 2020; Leidner & Neel, 2011; Thomas & Morris, 1994). Despite this rate of decline and the role of threatened invertebrates in essential ecosystem services such as pollination (Kim, 1993; Wagner & Van Driesche, 2010), there is still only a limited focus on their conservation around the world, including in Australia (Sands, 2018). Part of the problem resides in taxonomic issues, with many species undescribed and/or lacking basic taxonomic information (Hochkirch, 2016; Kim, 1993; New & Sands, 2004), leading to the risk that some species may face extinction even before they are known. Yet in Australia, many threatened invertebrates represent unique evolutionary lineages that form an important component of biodiversity (Cranston, 2010).

Although genetic data are critical in informing conservation strategies, helping to resolve taxonomic issues, defining patterns of connectedness across populations, and assessing the adaptive capacity of populations to future environmental changes, very little genetic data exist for threatened terrestrial invertebrate species. Older work using mtDNA, AFLPs, allozymes, microsatellites, and other markers has been used to define management units for conservation (e.g. Roitman et al., 2017; Rotheray et al., 2012), examine gene flow and historical processes (e.g., Crawford et al., 2011; Vogler et al., 1993), and explore the consequences of management actions such as insect translocations (Witzenberger & Hochkirch, 2008). There are so far relatively few attempts to integrate modern genomic approaches based on genome‐wide SNPs or genome resequencing into invertebrate conservation efforts (e.g., Chen et al., 2017; Dupuis et al., 2020). These approaches can provide very detailed information on patterns of gene flow, hybridization, and evolutionary potential in threatened species that can guide management actions (Allendorf et al., 2010).

Here we provide SNP and mtDNA‐based analysis of populations of an endangered morabine grasshopper, *Keyacris scurra* (formerly known as *Moraba scurra*). Morabines represent a unique group of Australian flightless grasshoppers, with a characteristic matchstick‐like appearance. The morabines consist of ~250 species and 41 genera found across Australia on a range of plant types including grasses, trees, and shrubs (Blackith & Blackith, 1969; Key, 1977). *Keyacris scurra* is one of the better‐known morabines. The genus *Keyacris* was named after the entomologist Ken Key (Day & Rentz, 2004) and studied by the Australian geneticist and evolutionary biologist Michael White (White, 1956; White et al., 1963). The species was used in pioneering work on adaptive genetic polymorphisms in collaboration with the American evolutionary biologist Richard Lewontin (e.g., Lewontin & White, 1960; White et al., 1963) which led to an ongoing debate about population processes affecting chromosomal polymorphisms and particularly the existence of adaptive landscapes (reviewed in Grodwohl, 2017). The species has two chromosomal races, defined through chromosome preparations from male genitalia as either 2*n* = 15 (seven pairs of autosomes and a small acrocentric X‐chromosome) or 2*n* = 17 (with one of the autosomes split into two acrocentric chromosomes) (White, 1956). Populations can also differ in the frequency of chromosomal arrangements that represent inversion polymorphisms (White, 1956).

The species was found in northeastern Victoria in the wheat/grazing belt and in the wheat/grazing belt of eastern New South Wales (NSW) as far north as Orange. White (1956) noted that *K. scurra* was already threatened when he indicated that they consist of “relatively minute ‘islands’ in the general area within which the species occurs.” Most of these "ecological islands" studied by White were places which had escaped agricultural intensification and regular grazing, such as small rural cemeteries, small reserves, and railway cuttings (Rowell & Crawford, 1995).

The species appears confined to habitats of a special type in which the tall perennial grass, *Themeda triandra,* usually predominates. This once dominant grass is removed by cropping and is grazing sensitive, and it now only dominates relict areas, which are often also refuges for other similarly sensitive plant species (Dorrough & Scroggie, 2008), including many daisies that *K. scurra* requires for food (White, 1956). Suitable habitats occur in grassland, savannah woodland, and on the ecotones between the latter habitats and both "dry" and "wet" sclerophyll forest. *Keyacris scurra* is an overwintering species, hatching in summer and with a univoltine life cycle. The species is unfortunately found within one of the most modified regions of Australia (Glanznig, 1995) where very little remnant habitat remains. The species has very limited dispersal ability due to its flightless habit. The main threat likely remains the management of vegetation (e.g., cemeteries are now managed by repeated mowing close to ground level which destroys the habitat of *K. scurra*).

Here we use genome‐wide SNP markers to assess the genetic structure of *K. scurra*, establish patterns of genetic variation in remnant populations, and consider associations between genetic variation and the extent of remaining suitable habitat. We resample areas where the species was previously found as well as new areas that appear to have suitable vegetation. Our results show a high level of genetic differentiation across regions even when these are relatively close together, evidence for genetic isolation by distance in both nuclear and mtDNA markers, evidence of inbreeding in some populations, and some genetic differentiation patterns unrelated to the chromosomal constitution of populations. We show that genetic diversity varies among populations, and we test whether this variability as well as inbreeding is related to the extent of habitat available in the proximity of the sampling sites. We find that even very small habitat patches may support populations with valuable genetic diversity, although increased inbreeding seems to relate to smaller habitat size.

## 2 METHODS

### 2.1 Sampling sites

**FIGURE 1:**
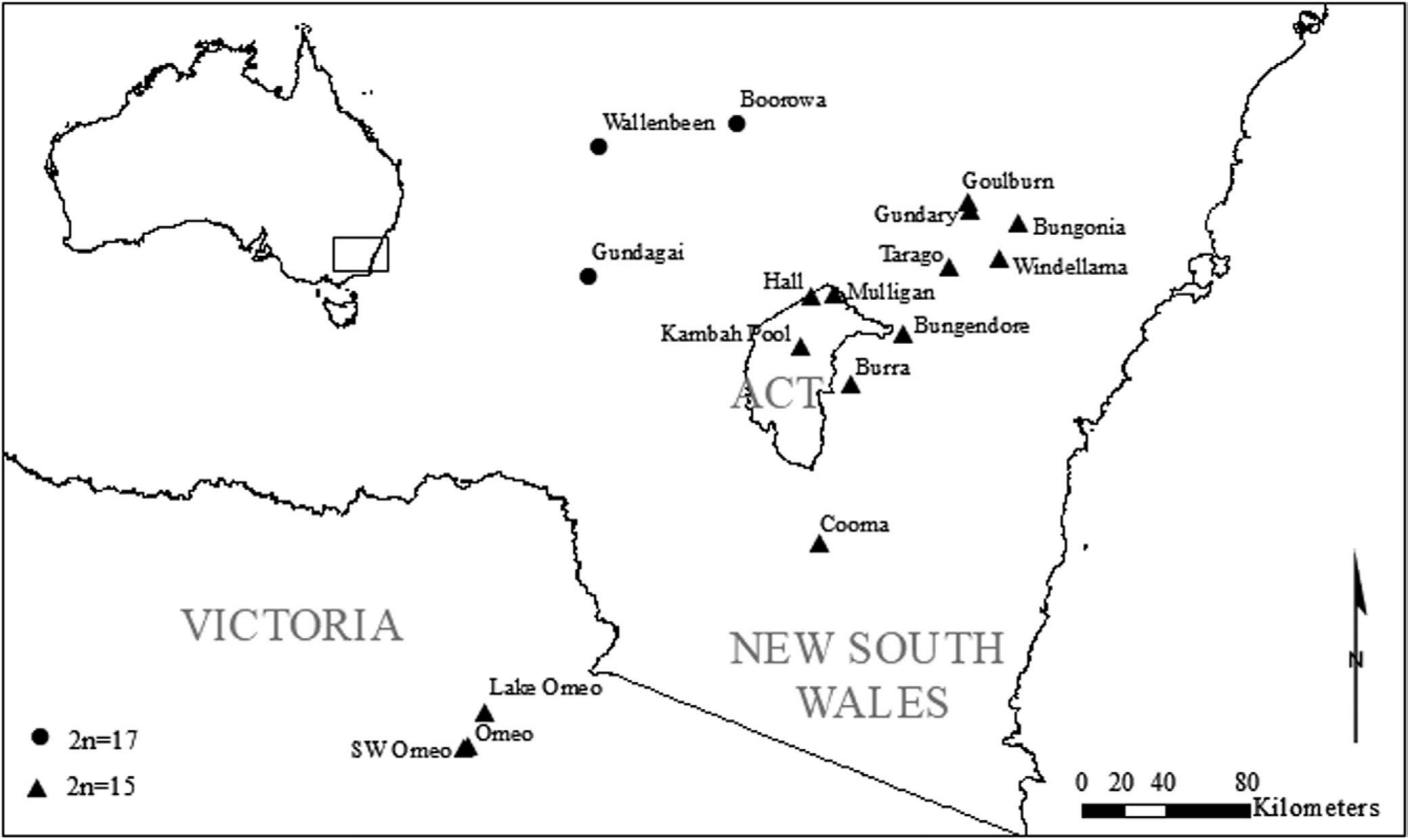
Map of sites surveyed for molecular variation. These sites encompass most of the current known fragmented distribution of *Keyacris scurra*. Singletons from two additional sites were included in the molecular survey: a site close to Gundagai (“Gundagai Cemetery”) and a site close to Windellama (“Windellama North”). Chromosomal races based on White (1956) are displayed in black dots (2*n* = 17) and triangles (2*n* = 15)

Samples of *K. scurra* were collected from 17 locations from NSW and Victoria in 2019 for molecular work following an extensive survey to map the current distribution of this species (Figure 1). These samples had been collected prior to the listing of the species as “endangered” in NSW (https://www.environment.nsw.gov.au/resources/threatenedspecies/determinations/CAMKeysMatchstickGrasshopperESPD.pdf) and with the approval of the Department of the Environment, Land, Water and Planning in Victoria following the rediscovery of the species from Omeo. Only a few individuals were collected from the smaller populations (particularly at Bungonia, Gundagai South Cemetery, Windellama North) (Table 1). Two sites (Windellama and Bungendore) had previously been subjected to a deliberate translocation by White (1957). Grasshoppers were collected with aspirators across an area of >20 m^2^ and preserved individually in 100% ethanol in Eppendorf tubes. They were then brought back to the laboratory for DNA processing.

**TABLE 1:** Populations and sample sizes included in genetic analysis of geographic variation

Population	*N*	Latitude	Longitude	Environment	Chromosomal race (2*n*, males)^a^	Collection rate (hoppers/person/min)	H_o_ (one SNP per sequence)	H_e_ (one SNP per sequence)	*F*_IS_ ((H_e_−H_o_)/H_e_)
Boorowa	19	−34.439	148.729	Open woodland	17	0.33	0.033	0.040	0.162
Bungendore	4	−35.342	149.429	Cemetery	15	0.06	0.030	0.028	−0.066
Bungonia	2	−34.863	149.942	Grassland verge	15	0.10	0.019	0.023	0.170
Burra	19	−35.552	149.229	Open woodland	15	0.50	0.027	0.029	0.055
Cooma	16	−36.234	149.093	Open woodland	15	0.28	0.016	0.016	0.035
Goulburn	20	−34.772	149.731	Open woodland	15	1.10	0.030	0.033	0.103
Gundagai South^b^	6	−35.096	148.091	Cemetery	17	0.32	0.038	0.037	−0.022
Gundagai Cemetery	1	−35.052	148.112	Cemetery	17	0.05	—	—	—
Gundary	2	−34.802	149.738	Open woodland	15	0.02	0.029	0.023	−0.265
Hall	21	−35.173	149.058	Open woodland	15	0.84	0.031	0.036	0.139
Kambah Pool	4	−35.395	149.012	Grassland	15	0.11	0.028	0.029	0.051
Lake Omeo	5	−36.964	147.657	Grassland verge	15	0.25	0.025	0.028	0.080
Mulligans	4	−35.166	149.155	Open woodland	15	0.17	0.032	0.030	−0.076
Omeo	3	−37.107	147.580	Open woodland	15	0.37	0.023	0.022	−0.055
SW Omeo	3	−37.115	147.561	Grassy verge	15	0.26	0.023	0.020	−0.115
Tarago	6	−35.046	149.654	Grassy verge	15	0.53	0.028	0.031	0.085
Wallendbeen	19	−34.405	148.258	Grassy railway cutting	17	0.33	0.034	0.037	0.090
Windellama	2	−35.014	149.863	Cemetery	15	0.07	0.029	0.024	−0.224
Windellama North	1	−34.976	149.900	Grassy verge	15	—	—	—	—

Note

Note that all sites except for two sites where singletons were sampled (Windellama North, Gundagai Cemetery) were included in the analysis of genetic variation within localities.

^a^ Based on White (1956), defined through chromosomal squashes on male genitalia.

^b^ We refer to this site as Gundagai in the analyses.

### 2.2 CO1 PCR and sequencing

A total of 59 individuals were screened from the ACT and NSW (13 populations, 43 individuals) as well as Victoria (3 populations, 16 individuals) with all regions in Table 1 represented. DNA was extracted using a Chelex^®^ 100 Resin (Bio‐Rad Laboratories, Hercules, CA) method on the upper half of a grasshopper hind limb. Tissue was crushed with 2 × 3 mm glass beads and 200 µl of 5% (w/v) Chelex^®^ 100 suspension using a mixer mill. Extractions were incubated for 2 hr at 60°C with 5 µl proteinase K (20 mg/ml) (Roche Diagnostics Australia, Pty Limited) and heated at 90°C for 10 min. Prior to polymerase chain reaction (PCR) amplification, extractions were spun at 14,000 rpm for 2 min, and DNA in solution was removed from just above the Chelex^®^ resin.

Polymerase chain reaction was performed to amplify approximately 700 base pairs (bp) of the mitochondrial cytochrome oxidase subunit 1 (COI) gene using the primer combination LCO1490: 5'‐ggtcaacaaatcataaagatattgg‐3' and HCO2198: 5'‐taaacttcagggtgaccaaaaaatca‐3' (Folmer et al., 1994). A 50 µl reaction volume was used with 0.2 mM dNTPs, 0.1 mg/ml bovine serum albumin (New England Biolabs), 1X reaction buffer, 2.0 mM MgCl_2_, 2 units of Taq polymerase (New England Biolabs), 0.20 µM forward and reverse primers, and 4 µl of 1:10 diluted template DNA.

The PCR amplification profile for COI consisted of an initial denaturing step at 95°C for 4 min (1 cycle), 40 cycles of denaturation at 95°C for 45 s, annealing at 53°C for 45 s and extension at 72°C for 1 min, and then a final extension step at 72°C for 5 min (1 cycle). All PCRs were conducted in Eppendorf Mastercycler S Gradient machines. PCR amplicons were sequenced from both directions using Sanger sequencing (Macrogen Inc.), and the chromatograms were analyzed using Geneious version 11.1.4 (http://www.geneious.com, Kearse et al., 2012).

### 2.3 DArT‐Seq™ processing

A high‐throughput genotyping method using the DArT‐Seq™ technology at Diversity Arrays Pty Ltd was employed. Here, complexity reduction is used to enrich nuclear genome representations with active genes and low copy sequences through combinations of restriction enzymes and reduction methods (https://www.diversityarrays.com/technology‐and‐resources/dartseq/ and Kilian et al., 2012). Implicit fragment size selection and next‐generation sequencing of representations are subsequently performed with HiSeq2000 (Illumina) (Georges et al., 2018; Kilian et al., 2012). This technology was considered appropriate for *K. scurra* to overcome sequencing problems associated with large genomes and high levels of repetitive DNA, gene duplications, and pseudogenes which were expected in this orthopteran species (e.g., Palacios‐Gimenez et al., 2020; Wang et al., 2014).

Grasshopper hind limb tissue (upper half) was supplied to Diversity Arrays Pty Ltd (Canberra, Australia) for high‐density (approx. 2.5 million sequences/sample used in marker calling) DArTSeq™ assay. Eight samples were first tested with multiple restriction enzyme combinations, and an “optimal” set was determined based on the fraction of the genome represented, while controlling average read depth and the number of polymorphic loci (https://www.diversityarrays.com/technology‐and‐resources/dartseq/). DArTSeq™ DNA extraction,sequencing and SNP genotyping methods are explained in detail elsewhere (Georges et al., 2018; Kilian et al., 2012).

### 2.4 Bioinformatics for nuclear data

#### 2.4.1 Read processing

Following adaptor and barcode sequence trimming, raw fastq files of DArTSeq™ samples (HiSeq processing) were processed with the STACKs denovo_map.pl pipeline (version 2.0b, Catchen et al., 2013), as there is no reference genome for *K. scurra*. This pipeline assembles loci de novo within each individual, combines these loci into a catalog, matches individuals to the catalog, and then performs SNP calling and haplotype phasing. Program settings were customized to allow four mismatches between sequence stacks within individuals (*M* = 4) and the same number between stacks between individuals (*n* = 4). Genotyped SNPs were outputted to the VCF file format for read filtering.

#### 2.4.2 Read filtering

Before further SNP filtering was carried out, one individual with low‐quality reads was removed from the dataset. To investigate the effects of sample selection and filtering assumptions on downstream measures of genetic diversity and differentiation, we created a range of differing datasets at the SNP filtering stage (see below) with VCFtools (Danecek et al., 2011). In all cases, only loci with <5% missing data across those individuals included within a dataset were retained.

Datasets were constructed by varying individuals per population, minor allele count (MAC) (in the case of heterozygosity), whether all SNPs or one SNP per sequence were included in the analysis, and whether all nucleotides including monomorphic nucleotides were included in estimates of heterozygosity. MAC rather than minor allele frequency was used, as recently advocated for population structure analysis (Linck & Battey, 2019). We used a different filtering approach when considering estimates of population variation (Schmidt et al., 2020) versus population structure. A minimum minor allele count of 3 was used when assessing population structure, given that this filter appears to be optimal for this purpose particularly when using programs like STRUCTURE (Linck & Battey, 2019). However, a MAC of 1 was used when characterizing variability within populations. While researchers normally use the same filter when characterizing population structure and variation within populations, this can lead to biased estimates, as can filtering without considering differences in sample size between populations (Schmidt et al., 2020). We therefore filtered with 3 randomly sampled individuals per population (2 in the case of three of the populations where 3 individuals were not available, see Table 1) but using the SNPs identified from this filtering process, we then computed heterozygosity for all individuals. Following the recommendation in Schmidt et al. (2020), we also computed heterozygosity based on all nucleotides (i.e., including all polymorphic and monomorphic nucleotides).

#### 2.4.3 Heterozygosity

In characterizing individual heterozygosity, we derived three estimates; heterozygosity was computed based on either one SNP per (80 bp) locus randomly selected from the datasets, all SNPs across sequences, or all nucleotides sequenced including those that were monomorphic. In the first approach, there were 2060 SNPs retained from 13,518 SNPs identified at MAC = 1 for the heterozygosity estimate, whereas when all SNPs were retained, there were 10,618 SNPs that contributed.

All datasets were passed to the R “adegenet” package as genind objects for further calculations of heterozygosity and other statistics. Sites with only one individual were excluded from population measures but were included in individual heterozygosity assessments. The “Hs” function from “adegenet” was used to calculate expected heterozygosity for populations.

#### 2.4.4 Population structure analyses

The “all individuals” and “MAC = 3” dataset was used as the basis of a run of the program STRUCTURE in its ADMIXTURE mode (Pritchard et al., 2000). In these analyses, we did not want to bias toward variable regions and therefore we filtered by randomly selecting only one SNP per sequence. Following inference of lambda, MCMC runs were completed with a burn‐in of 10,000 and a further 100,000 repeats for parameter inference. *K* values between *K* = 1 and *K* = 10 were investigated, with ten runs per value of *K* and *K* = 10 set as the maximum based on available computing resources. Results were passed to the program CLUMPAK for collation and summarizing and evaluated according to various K‐inference procedures to determine optimal settings (Kopelman et al., 2015), with *K* = 6 being selected by the modified Evanno method. A further run was conducted with the “even populations” and “MAC = 3” dataset, under the same conditions and a separate lambda inference.

Datasets with all individuals were passed through PCoA, PCA, and DAPC multivariate analyses (via “ade4” [Dray & Dufour, 2007] and “adegenet” [Jombart, 2008]). For principal component analysis, missing data were handled wherever possible by interpolation with the mean of the sampling location where the sample with missing data was found. The same principle was applied for the DAPC analyses.

#### 2.4.5 AMOVA

A hierarchical analysis of molecular variance (AMOVA) was conducted on the SNP dataset using the “pegas” amova function implemented in “poppr” (Kamvar et al., 2014) for three levels: (a) individuals, (b) sites, and (c) regions. This last level was defined to include 6 regions: Cooma, Omeo, northern ACT (Mulligans, Hall), west (Gundagai, Wallendbeen, Boorowa), northeast (Tarago, Windellama, Bungonia, Gundary, Goulburn), and southeast (Bungendore, Kambah Pool, Burra). 1,000 permutations were conducted to test for significance across the differing levels.

#### 2.4.6 *F*_ST_ and isolation by distance based on SNPs

Pairwise *F*_ST_s for each population were calculated via the “pairwise.WCfst” function in the R package “hierfstat” (Goudet, 2005). These were converted to a distance measure, $\frac{F_{\text{ST}}}{\left(1‐F_{\text{ST}}\right)}$ and compared to geographic distance between sites (in km) to check for isolation by distance. Mantel tests comparing distance matrices and genetic distance were also undertaken in “adegenet.”

### 2.5 Phylogenetic analysis

To provide another approach to look at associations among populations, phylogenetic relationships among the populations were established using dartR, version 1.1.6 (Gruber et al., 2018) within the R programming environment (version 4.0.3, R Development Core Team, 2018). For DArTSeq™ nuclear data, we concatenated (a) sequence fragments (trimmed sequence tags with SNPs) and (b) SNPs only across loci for individuals where heterozygous positions were replaced by the standard ambiguity codes, and exported these as FASTA files. There were 5,608 SNPs retained after the Diversity Arrays filtering used prior to the phylogenetic analysis. Sequences were aligned and checked in MEGA, version 5.2 (Tamura et al., 2011), and SNP data were then converted to a NEXUS file format using the "ape" R package, version 5.4 (Paradis & Schliep, 2019). The FASTA file and NEXUS file were imported into CIPRES Science Gateway, version 3.3 (https://www.phylo.org/) (Miller et al., 2010) for maximum‐likelihood (ML) and Bayesian inference (BI) phylogenetic analyses, respectively.

For ML, we used the program RAxML, version 8.2.12 (Stamatakis, 2014) on the “sequence fragments,” that is, variant plus invariant data, for improved branch length and topological accuracy in phylogenetic trees (Leaché et al., 2015). We assessed support for the best ML topology by performing 504 nonparametric bootstrap (BS) replicates using the autoMRE option with the GTR GAMMA site‐rate substitution model. For BI, we used the program Mr Bayes, version 3.2.7a (Ronquist & Huelsenbeck, 2003) on the variant SNP data because of computational time constraints when dealing with variant + invariant data. We avoided uncertainty about what substitution model to use by sampling across the entire general time reversible (GTR) model space (“nst = mixed”) and chose a “proportion of invariable sites + gamma” model of rate variation (“rates = invgamma”) because this works well for many datasets (http://mrbayes.sourceforge.net/mb3.2_manual.pdf). Four independent Monte Carlo runs each with four Markov chains (MCMC) were done for 20,000,000 generations using random starting trees and a temperature parameter value of 0.1. Trees were sampled every 500 generations, and the first 25% of generations were discarded as burn‐in. The MCMC trace files were visualized and analyzed in the program Tracer, version 1.7.1 (Rambaut et al., 2018).

The best‐scoring ML tree and consensus BI tree were imported into FigTree, version 1.4.4 (http://tree.bio.ed.ac.uk/software/figtree/) for incorporation of branch length and support value (BS for ML and probability for BI) information. Resultant files were then visualized using the R packages ggtree, version 2.0.4 (Yu et al., 2017), ggplot2, version 3.3.2 (Wickham, 2016), and treeio, version 1.10.0 (Wang et al., 2020) packages.

### 2.6 mtDNA analysis

For all CO1 coding sequences, we first performed amino acid translations and searches for premature stop codons in Geneious version 11.1.4 (http://www.geneious.com) and confirmed sequence identity using BLASTn sequence homology searches against the National Center for Biotechnology Information (NCBI) nonredundant nucleotide database (https://blast.ncbi.nlm.nih.gov/Blast.cgi?PROGRAM=blastn&PAGE_TYPE=BlastSearch&LINK_LOC=blasthome). A haplotype network was generated using PopART version 1.7 (http://popart.otago.ac.nz) and the minimum spanning network option. This program was considered appropriate because we had no sites with missing data.

Genetic and geographic distance matrices were created using the average number of base pair differences and latitude and longitude coordinates respectively for all pairwise population comparisons. A relationship among distance measures was investigated using Mantel tests performed with the “mantel.randtest” function in R package “ade4” (Dray & Dufour, 2007) with 1,000,000 permutations. Nuclear genetic distance was also compared to mtDNA distance with nuclear distance calculated as the Euclidean coancestry coefficient.

### 2.7 Vegetation analysis

We assessed whether genetic variation is related to the area of available habitat, measured at different scales. Such an association might be expected given that *K. scurra* is closely associated with relict patches of *Themeda* grasslands through its basic requirements for food and shelter (White, 1956). To analyze the extent of available suitable habitat at each collection point, we compared between the raster of the National Vegetation Information System (NVIS) Version 5.1, a High‐resolution Satellite Imagery, and a pre‐existing likelihood model of intact native grassland (hereafter “grassland model,” S. J. Sinclair and M. D. White, unpublished). This last one was selected for the final analysis because its projection of 25 × 25 m cell size, compared to the 100 × 100 m of the NVIS raster, made it more concordant with the scale of our study. The grassland model was originally built for the Victorian State Government, and it was extrapolated to cover sites in NSW (grassland model details provided in Supplementary Material).

Given the low vagility of the *K. scurra*, we considered available habitat within radii 25, 50, 100, 250, and 500 m around each collection point using the ArcGIS 10.6 buffer tool. Available habitat was quantified by the sum of the pixel values of the grassland model within the relevant radius (Supplementary Material). The computed available habitat measures were then correlated with the observed heterozygosity and inbreeding (*F*_IS_) determined from heterozygosity estimates where MAC = 1 and an original filtering step where an equal number of individuals per population were maintained.

## 3 RESULTS

### 3.1 Population variation

**FIGURE 2:**
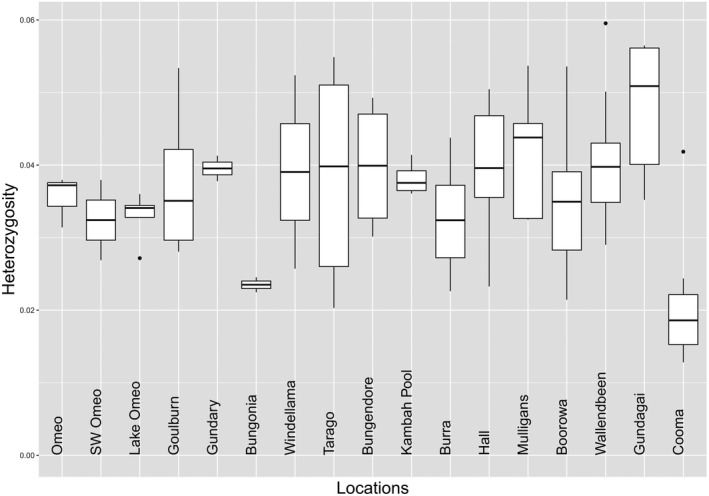
Box plots for individual heterozygosity by location (for selected SNPs, all individuals prefiltered to MAC = 1 from data where population with an even number of individuals were sampled, but then computed for all individuals from a population). Note that populations are ordered to match the STRUCTURE analysis below. Gundagai Cemetery and Windellama North are not included here because they are represented by singletons

The three measures of heterozygosity variation we estimated for populations based on individuals were all highly correlated across populations (*r* > 0.92). We therefore ran all further analyses with one SNP retained per sequence with all individuals considered, which maximizes the retention of variation in populations where sample sizes are small, but nevertheless allows all individuals from a population to be used in computing heterozygosity (Figure 2 and Table 1).

There was a significant difference in heterozygosity among populations when heterozygosity of individuals was compared (*p* < 0.001, Figure 2). Observed population heterozygosity (H_o_) varied from expected heterozygosity (H_e_) in some cases as reflected by *F*_IS_ computed as ((H_e_−H_o_)/H_e_), resulting in some *F*_IS_ values that were substantial and positive (e.g., Boorowa) (Table 1). These results suggest inbreeding in some populations. At the population level, there was nevertheless a strong correlation between the observed and expected population heterozygosity (*r* = 0.89).

Cooma and Bungonia had particularly low levels of heterozygosity (Figure 2). The low level of heterozygosity at Bungonia may partly reflect inbreeding given the large *F*_IS_ although the sample size for this population was small (Table 1). While cemeteries may house persistently small populations, the sample from the cemetery at Gundagai South had a high level of heterozygosity (Figure 2) and populations not from cemeteries such as Omeo also had relatively low heterozygosity despite not showing inbreeding. Tarago was noteworthy in showing high variability in heterozygosity estimates which may reflect the inclusion of some inbred individuals (Figure 2). Overall, these patterns suggest a range of genetic variability levels in *K. scurra* populations and high levels of variation even in some populations where suitable habitat appears limited.

### 3.2 Vegetation associations

**FIGURE 3:**
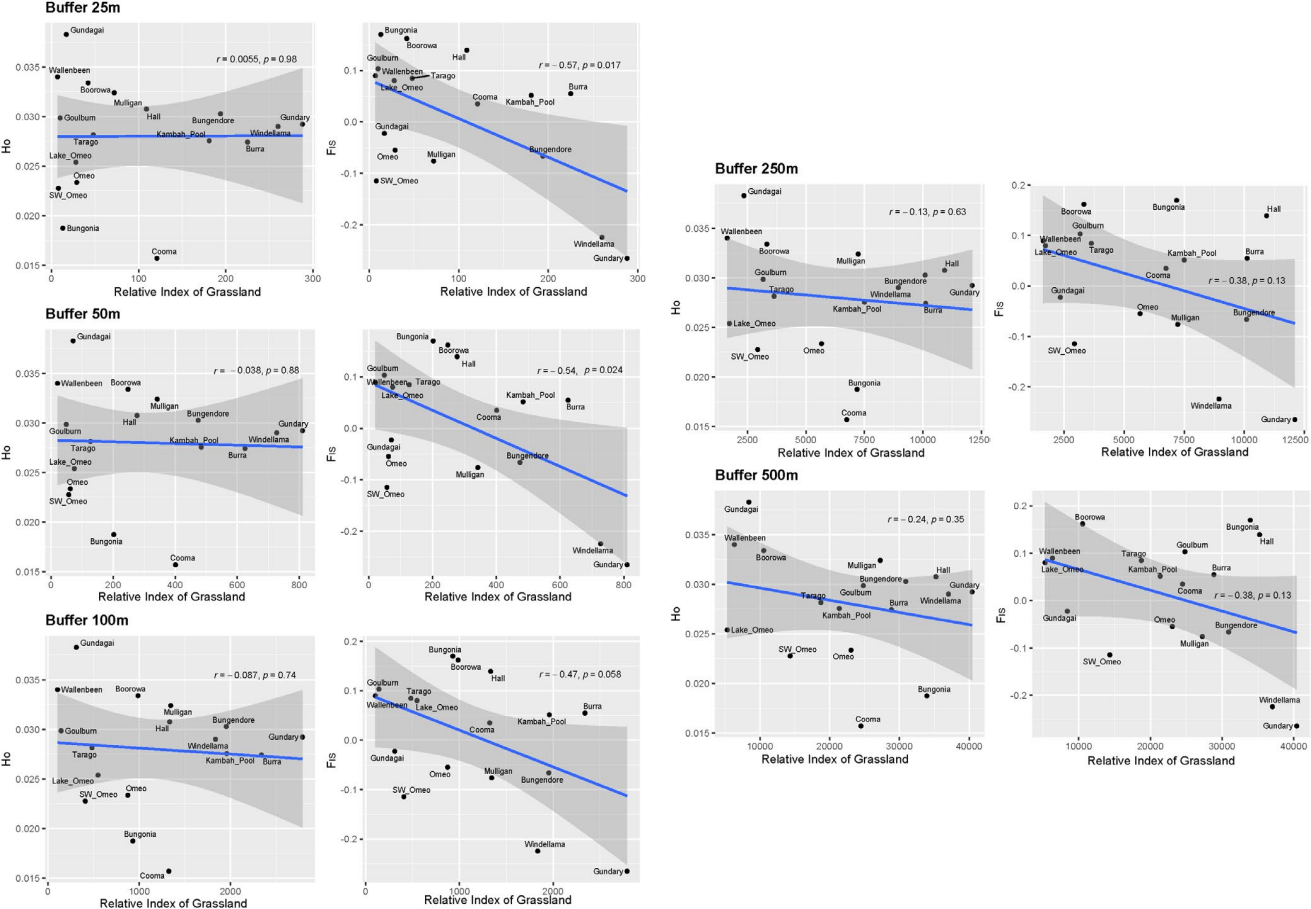
Association between a likelihood model of intact native grassland (S. J. Sinclair and M. D. White, unpublished) at different spatial scales (“buffers”) and observed heterozygosity (left column) or *F*_IS_ (right column). Dots reflect individual sites and are presented with correlation coefficients (*r)* and *p* values

We found a significant negative relationship between habitat area (buffered at 25 and 50 m) and *F*_IS_ (*r* = −0.54 and 0.57, *p* < 0.05), suggesting that populations in smaller habitat patches are more inbred (Figure 3, right column). This relationship was stronger when smaller radii were used to define available habitat. Relationships with observed heterozygosity were not significant (Figure 3, left column).

### 3.3 Population structure

#### 3.3.1 mtDNA

**FIGURE 4:**
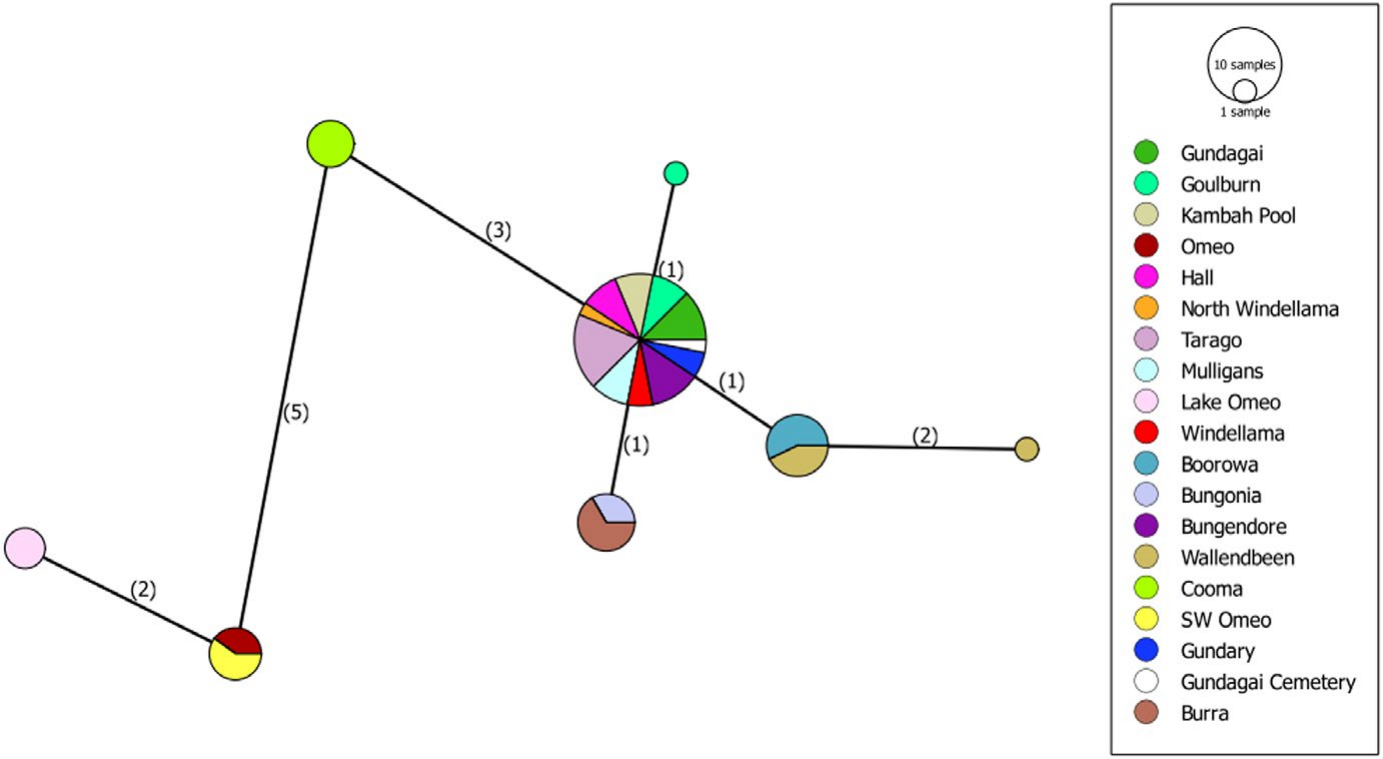
Variation in the COI gene sequence across *Keyacris scurra* as depicted by a network diagram. The numbers of nucleotide changes are indicated in brackets. The size of the colored areas reflects the number of haplotypes, and branch lengths reflect the number of nucleotide changes. COI, cytochrome oxidase subunit 1

The network diagram of mtDNA variation (Figure 4) indicates clear separation of the three Omeo sites from the other populations, with Cooma falling in between them and the other populations. For the remaining populations, two of the 2*n* = 17 populations (Wallendbeen and Boorowa), as determined from the earlier cytological work, fall apart from most of the populations but the other 2*n* = 17 sites (Gundagai South, referred to here as Gundagai, and Gundagai Cemetery) are not separated from the 2*n* = 15 populations, while two other 2*n* = 15 populations (Bungonia, Burra) also show some separation.

#### 3.3.2 SNPs

**FIGURE 5:**
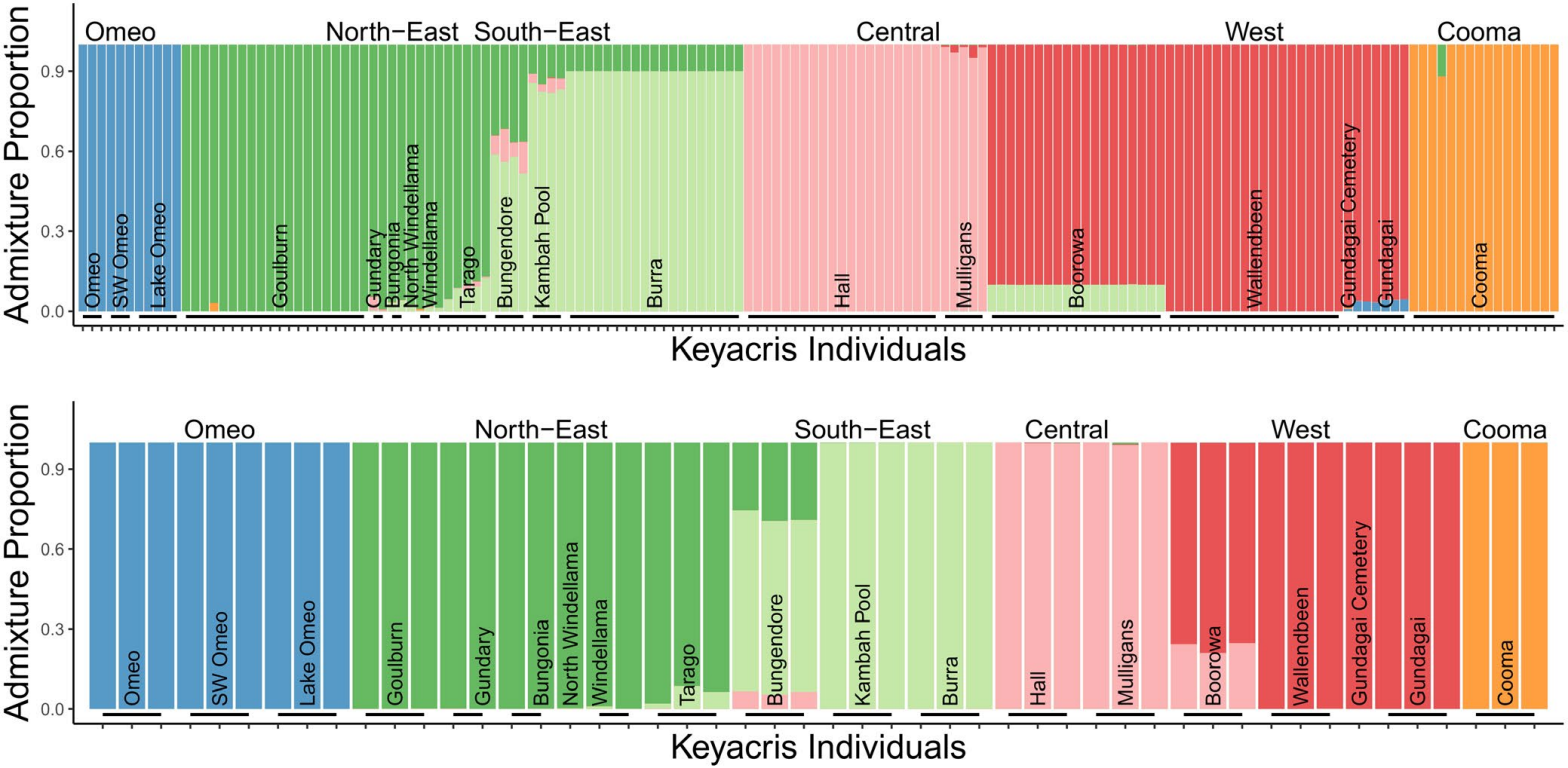
STRUCTURE plots for (a) all individuals and (b) even populations at *K* = 6 (best supported by modified Evanno method)

**FIGURE 6:**
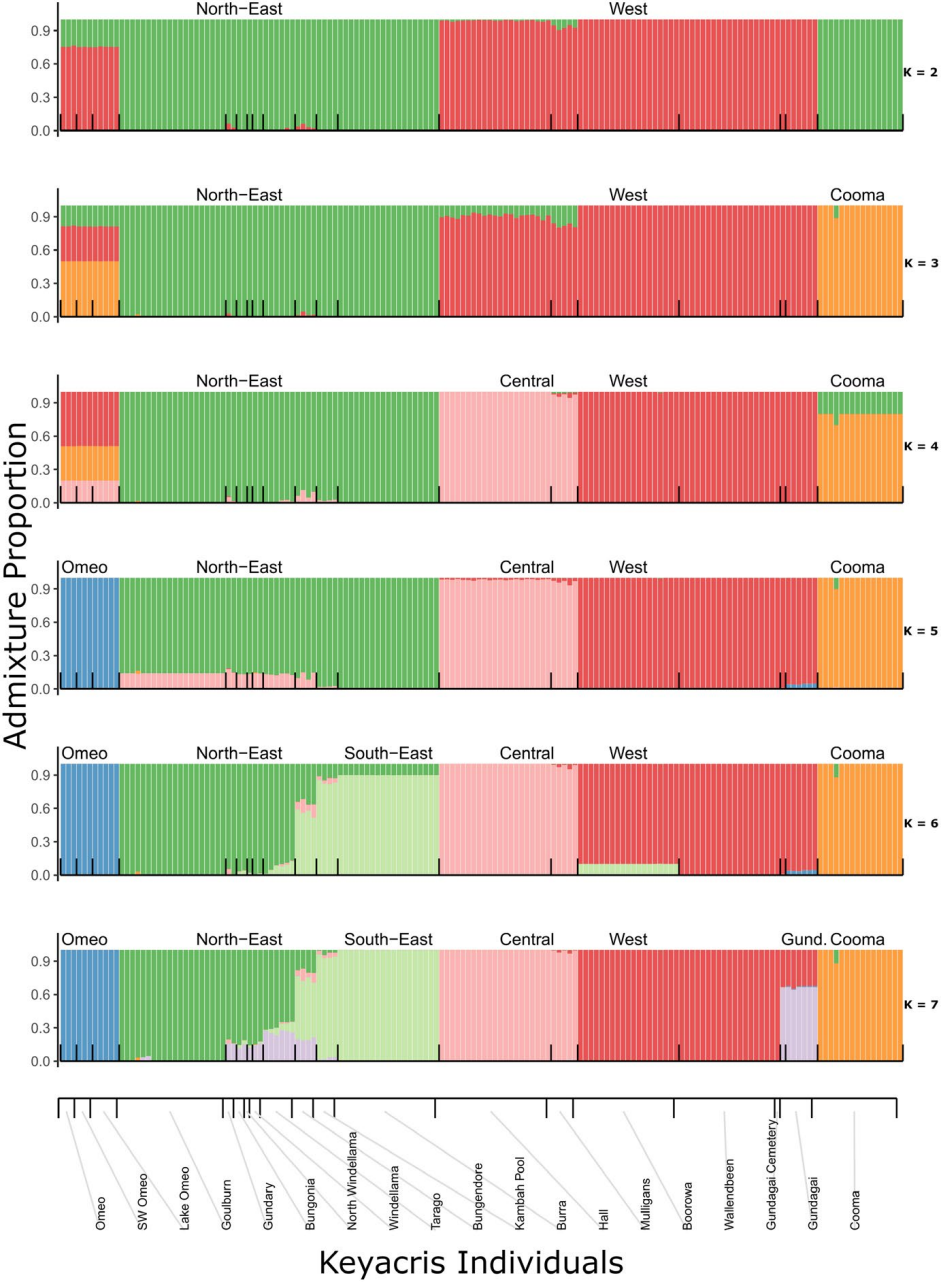
STRUCTURE analysis on all individuals from populations at different *K* values

The STRUCTURE plot for all individuals shows clear differentiation of regions at *K* = 6, separating the northeast, southeast, west, and central regions as well as distinguishing the outlying Cooma and Omeo populations (Figure 5). These patterns were clear regardless of whether all individuals were included in the analysis or whether an even number of individuals were selected from each population. Additional differentiation among sites was evident as *K* values were increased (Figure 6). An AMOVA (Table 2) indicated significant effects of regions and sites: 32% of variance is found within sites, 17% between sites within regions, and 51% between regions.

**TABLE 2:** Hierarchical AMOVA on SNP variation investigating the effects of region and site on molecular differentiation

Source	SSD	MSD	*df*	Estimated variance	Proportion	*p*
Region	12,626	2,525	5	82.326	0.506	<0.001
Site	2,792	214	13	28.124	0.173	<0.001
Within site	7,255	52	139	52.197	0.321	
Total	22,673	144	157	162.785	1.000	

**FIGURE 7:**
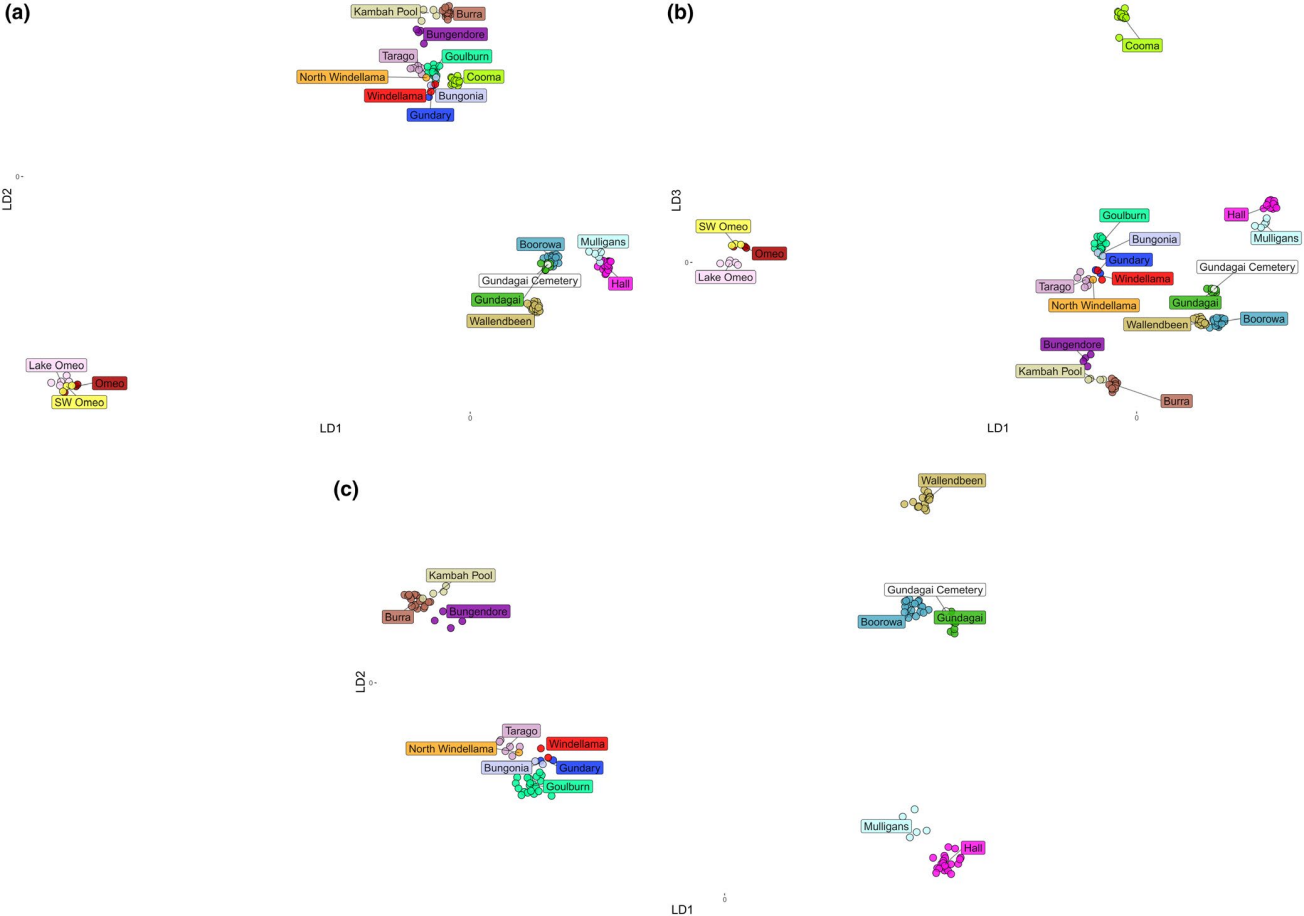
DAPC of *Keyacris scurra* individuals with Omeo and Cooma included along the two main linear discriminant (LD) axes (a) and the first and third axes (b) and when these populations are excluded (c) (*N* = 158, 45 PCs, RMSE 0.043 when all sites included; *N* = 131, 30 PCs, RMSE = 0.029 when Cooma and Omeo excluded)

**FIGURE 8:**
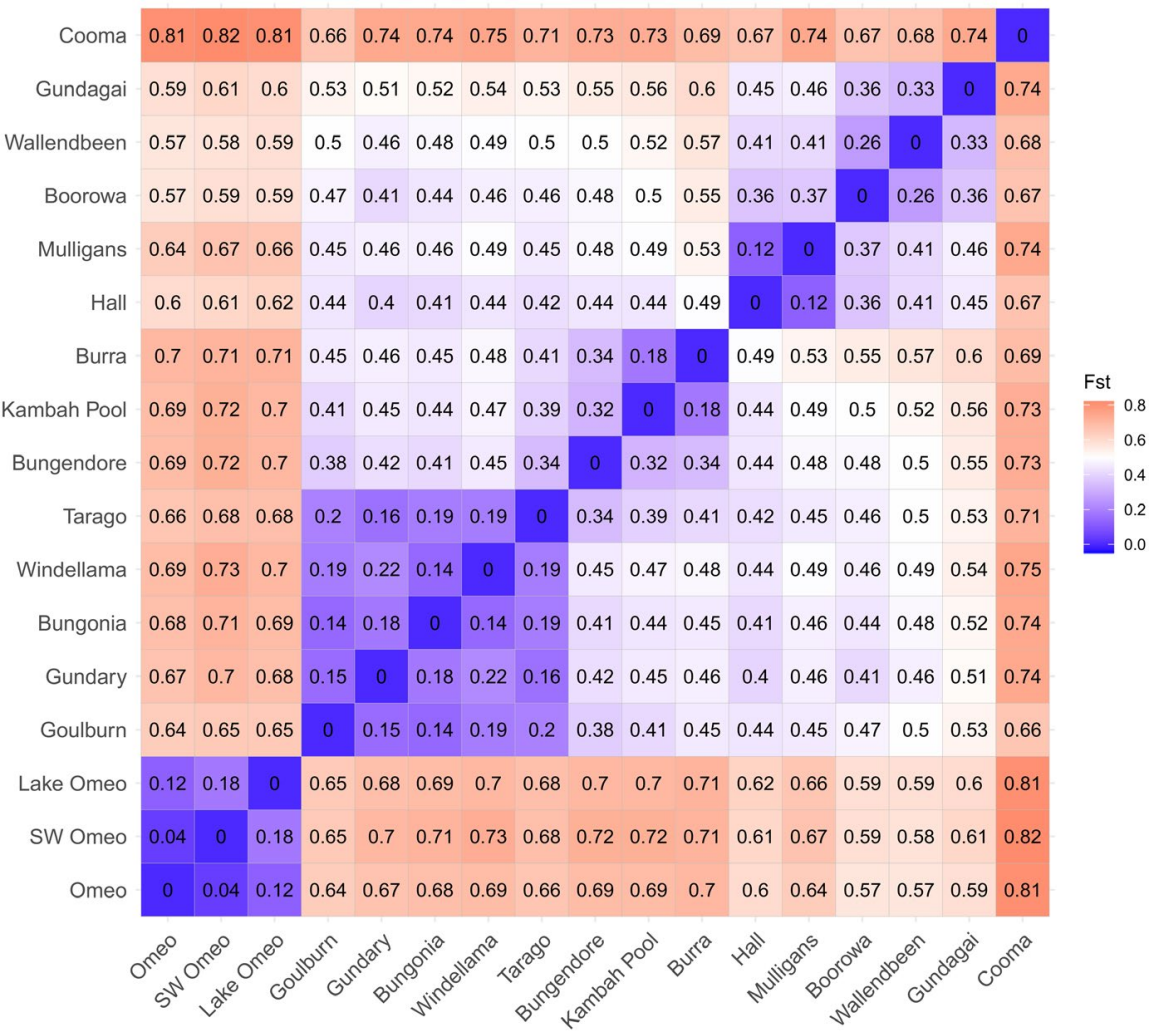
Pairwise *F*_ST_ values for comparisons of sites (excluding those with singletons)

The DAPC analysis provided a clear picture of differentiation that matched the results of the STRUCTURE analysis. When all individuals and sites were included, there was a strong separation of the Omeo populations from the other areas across the two main axes which accounted for 32% and 27% of the variation, with the other sites falling into two main groups (Figure 7a). Omeo and Cooma could both be separated based on the third axis (accounting for 17% of the variation) from all other populations (Figure 7b). Note also how individuals from the same site tend to clump close together even when they are all in the same region. When the Omeo and Cooma populations are excluded, patterns for the other regions become clearer (Figure 7c), with close associations between the ACT sites (Mulligans, Hall) and the 2*n* = 17 NSW populations (Boorowa, Wallendbeen). Based on the nuclear markers, the 2*n* = 17 Gundagai samples also fall close to the other 2*n* = 17 populations, unlike for the mtDNA markers. Apart from the Omeo and Cooma populations, the other 2*n* = 15 populations fell into two groups, but most individuals could still be allocated to sites, highlighting substantial differentiation across the sample sites even when these were quite close together. However Kambah Pool and Burra fell close together, as well as Windellama, Bungonia, and Gundary, (Figure 7). The *F*_ST_ values between sites (Figure 8) were variable and in many cases substantial, being around 0.8 for comparisons with Omeo and Cooma populations and varying within the range 10%–20% for the other populations. These substantial differences point to populations at sites that are often unique in terms of their nuclear composition even if there is overlap in mtDNA variation.

### 3.4 Phylogenetic analysis

**FIGURE 9:**
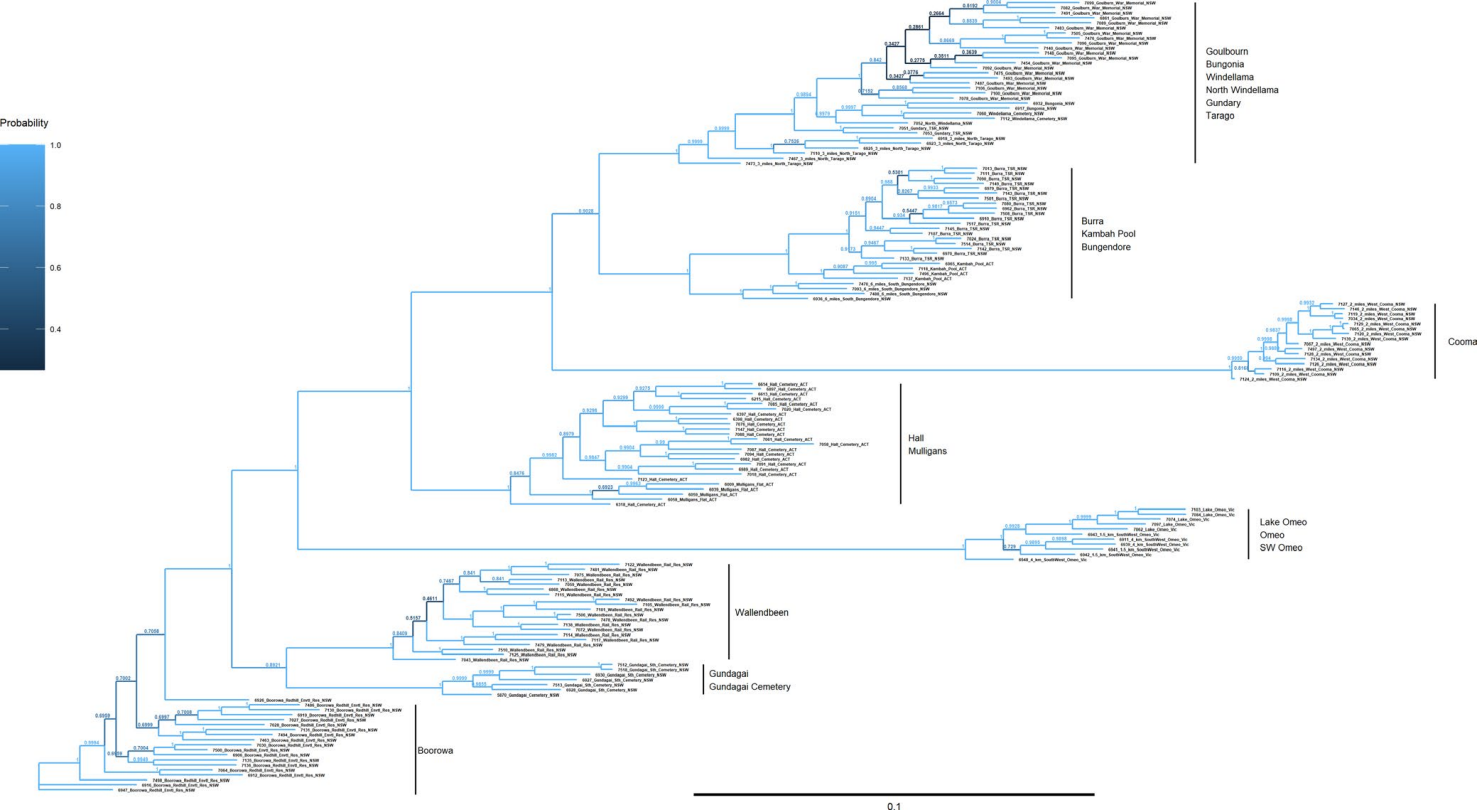
Bayesian tree representing individual grasshoppers generated from SNP data with probability support values shown at branch nodes. Note the tight clustering by site location

**FIGURE 10:**
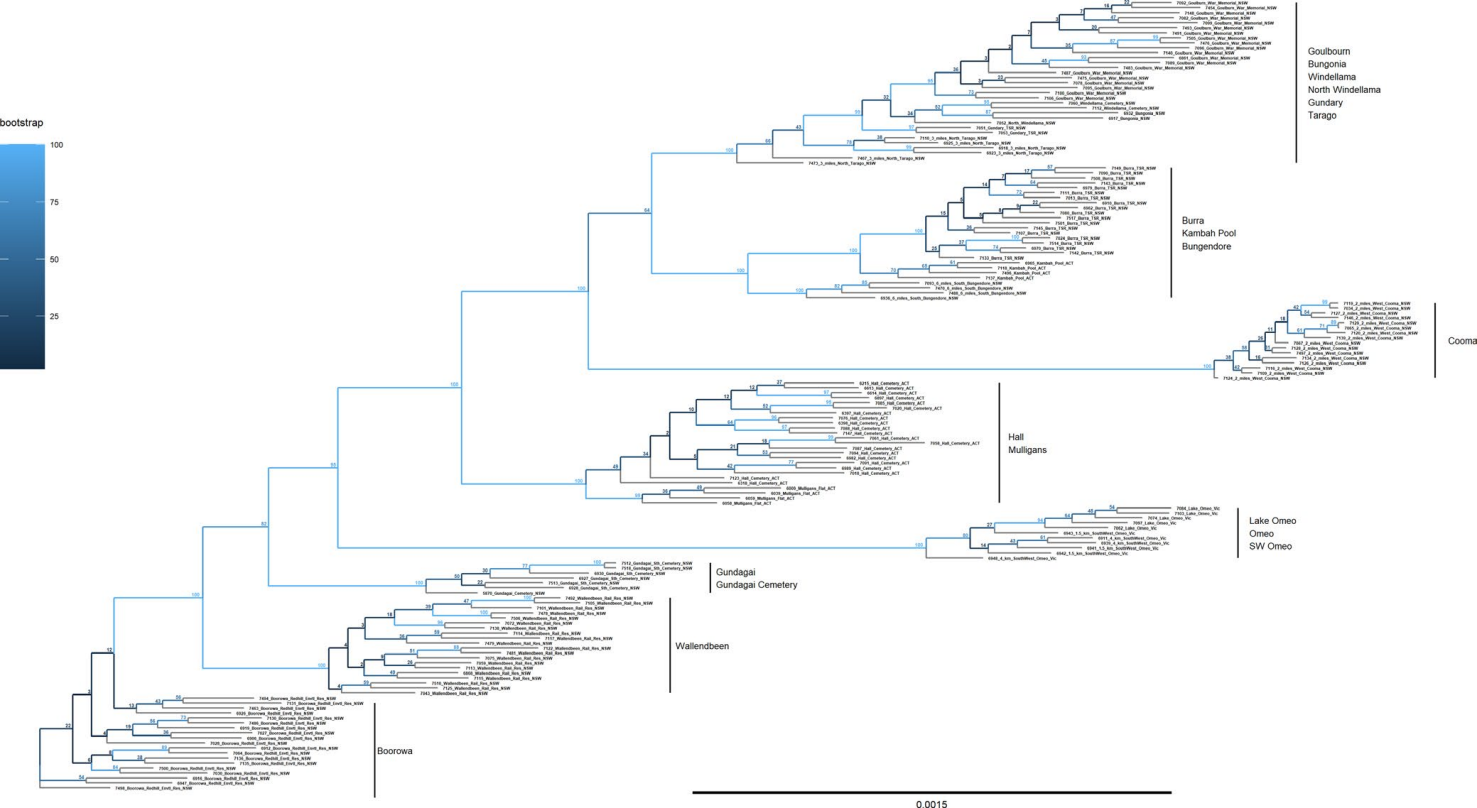
RAxML tree representing individual grasshoppers generated from SNP data with bootstrap support values shown at branch nodes. Note the tight clustering by site location

The phylogenetic analysis confirmed the uniqueness of the populations (Supplementary Information). Both the Bayesian tree (Figure 9) and the ML tree (Figure 10) showed that the individuals clustered into their collection sites. This included collection sites where the DAPC analyses did not clearly separate individuals into the collection sites, such as Kambah Pool and Burra, and also Windellama, Bungonia, and Gundary. Overall, the structure produced by these unrooted trees was consistent regardless of whether a ML analysis or a Bayesian analysis was used (Figures 9 and 10) with reasonable support for the clusters that were identified.

### 3.5 IBD analysis

**FIGURE 11:**
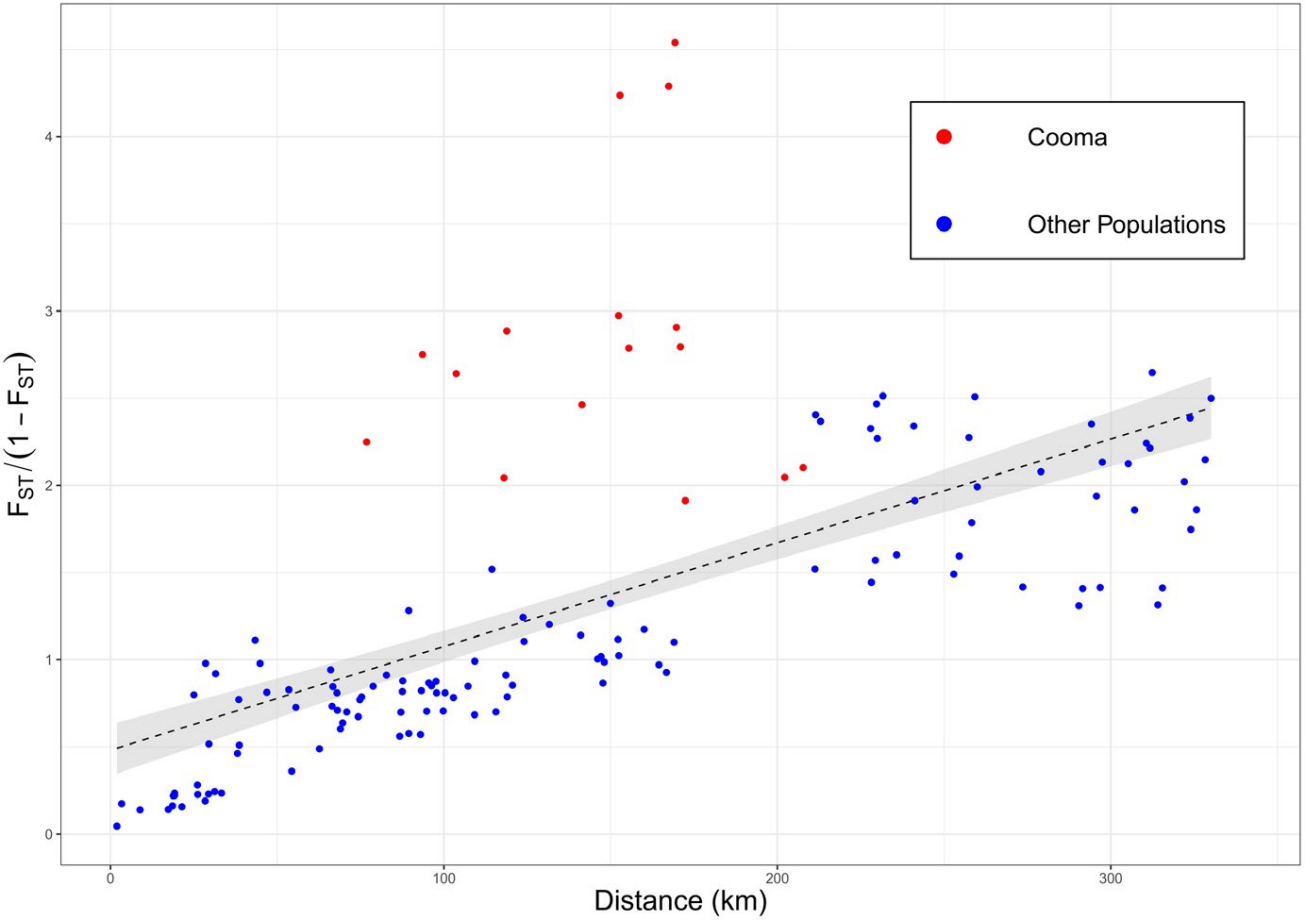
Correlation of geographic distance with *F*_ST_‐derived distance between populations

For analyses of IBD, *F*_ST_‐derived distance (*F*_ST_/(1−*F*_ST_) was regressed against geographic distance (Figure 11) with the relationship being highly significant (*p* < 0.001, *R* ^2^ 0.7729, slope 0.0059). Comparisons with the Cooma population were notable for falling above the line established from the other population comparisons, which is consistent with the high *F*_ST_ values for comparisons with this population (Figure 11). A Mantel test indicated a significant association between geographic and genetic distance (*r* = 0.7660, *p* < 0.001, 1,000,000 permutations) consistent with the IBD regression analysis.

We also ran an IBD analysis on the mtDNA data by comparing the number of nucleotide differences between populations. A Mantel test on the mtDNA data indicated a significant association between geographic distance and nucleotide differences (*r* = 0.876, *p* < 0.001) in agreement with the nuclear comparison. A Mantel test also indicated a positive association between the nuclear differences among populations and the mtDNA differences (*r* = 0.693, *p* < 0.001).

## 4 DISCUSSION

*Keyacris scurra* is an endangered species persisting for many decades in small areas where suitable habitat has remained. The grasshoppers from Windellama and Gundagai South cemeteries were sampled from suitable habitat covering only a few hectares which are surrounded by farmland. *Keyacris scurra* has persisted at these small sites since the 1950s and 1960s. Samples from both these sites show high levels of genomic variability relative to other samples, which suggests that there has been limited loss of genetic variation through genetic drift to date from these isolated sites. On the other hand, *K. scurra* has been lost from many other small remnant areas where they were recorded in the 1950s and 1960s, most likely through inappropriate site management. For instance, White et al. (1963) performed evolutionary studies on the cemetery site at Murrumbateman in the ACT, where we failed to find the grasshopper despite multiple attempts to locate them there. We also visited many cemeteries in Victoria where *K. scurra* had been present in the 1950s (White, 1956), but specimens could not be found. In these areas, we found that *T. triandra* grassland has often persisted, but we believe that site management has removed the specific habitat elements required for *K. scurra* to persist, either via the exclusion of daisies through overgrowth of *Themeda* (Stuwe & Parsons, 1977) or via structural modification of the grass sward by regular mowing to keep cemeteries neat (Clayden et al., 2018). These observations show how tenuous survival can be for threatened species in agricultural landscapes and how much they are subject to stochasticity and the unintended consequences of small‐scale management decisions. But they also indicate how well insect populations can survive in fragments as long as suitable habitat is available (Tscharntke et al., 2002).

The populations at Windellama and Bungendore had previously been subjected to a deliberate translocation by White (1957) who introduced males from other populations in an attempt to alter the chromosomal constitution of the populations to explore the potential effects of natural selection on chromosome polymorphisms. Translocations are expected to boost genetic diversity and result in hybrid populations that are genetically distinct from parental populations as noted for the threatened field cricket, *Gryllus campestris* (Witzenberger & Hochkirch, 2008) and adders (Madsen et al., 1999). Here we find that the two populations map in multivariate space with nearby populations (Figure 7) which were 19+ km away (Figure 1) despite being separated from that site by unsuitable farmland, suggesting that the past deliberate translocation in this case had little impact on the uniqueness of the natural population nor boosted genetic variation. However, there was a signal of hybridization from the STRUCTURE plots in the Bungendore population which would be worth exploring further.

At this stage, there is little support for the need to “genetically rescue” most populations of *K. scurra* from low levels of genetic variation within populations, perhaps with the exception of Bungonia and Cooma which had low variation and/or showed relatively high inbreeding. Genetic rescue involves the deliberate introduction of individuals across populations to overcome the deleterious effects of mutations that have become fixed in small populations (Weeks et al., 2011; Whiteley et al., 2015); it can be useful where there is strong evidence of a decline in genetic diversity and has been proposed as a useful approach for some threatened Australian insects (Roitman et al., 2017). However, with genetic variation persisting so far even in small areas, there is likely to be limited benefit from such an exercise. Instead, we suspect that it is important to maintain the remaining variation across the range of the species given that there is very strong genetic differentiation among the populations. The *F*_ST_ values of up to 0.8 are extremely large and imply that populations often have different alleles predominating at loci that are polymorphic even within the same chromosomal form. Both selection and genetic drift may have contributed to this high level of differentiation. Thus, our high‐resolution genetic data mirror differences in the frequency of chromosomal inversion polymorphisms in populations observed by White (1956) which varied among populations even when these involved the same chromosomal races.

The value of small reserves in preserving invertebrates (Hafernik, 1992) and plant biodiversity (Kendal et al., 2017) has been well recognized. However, small populations from reserves may lack genetic diversity which is linked to the adaptive capacity of populations (Hoffmann et al., 2017; Willi et al., 2006). In the case of *K. scurra* populations from restricted sites, like the cemeteries assessed here, the high level of diversity still remaining at these sites suggests that they may, at least for now, be able to counter environmental changes threatening populations into the future through evolutionary responses. Thus, while declines in invertebrate populations may well compare to those seen in plant and vertebrate populations (Leidner & Neel, 2011), remedial action to counter declines could be much easier through the recreation of small habitat areas. Habitats where *K. scurra* persist are quite variable and these will need to be managed in different ways to conserve *K. scurra*. For example, fire management practices could be modified to avoid burning or at least to reduce controlled burns during at‐risk life stages. During drought, browsing mammals (including native species) may need to be excluded to avoid overgrazing of *Themeda*. And cemetery management groups could be consulted to ensure that suitable habitat is fenced and not regularly mowed. Management of the Omeo populations will be particularly important since these appear to comprise the last remaining stronghold of the species in Victoria and are genetically quite distinct.

Our data on the associations between genetic variation and the area of available habitat are difficult to interpret without further investigation. We found a significant negative relationship between habitat area and *F*_IS_, indicating elevated breeding between related individuals in smaller sites, but no association with genetic variation as measured by heterozygosity. A naïve expectation would be that observed heterozygosity would reveal the opposite: a positive relationship with reduced heterozygosity at smaller sites. Our data did not show this trend; this unexpected relationship may be an artifact of our relatively small sample size and method of habitat measurement, but it may also have biological foundations, and reflect past expansions and contractions in the distribution and population size of *K. scurra*. For example, rapid postglacial expansion from refugia may have led to populations with a high residual heterozygosity, but a recent history of population fragmentation may be contributing to inbreeding in some populations. The high levels of observed heterozygosity compared to expected heterozygosity in some populations also warrant further investigation, particularly in relation to inversion polymorphisms which can directly affect heterozygosity (Kennington et al., 2006). Patterns in current populations may display the legacy of past events and ongoing chromosomal dynamics which could be resolved by additional genomic resources so that (for instance) comparisons of heterozygosity could be made within and outside of inverted regions and population histories could be documented from linkage data.

Why we failed to correlate apparent habitat mapping and levels of SNP variation is unclear. Apart from the grassland model, we did attempt several other approaches such as using polygons from satellite images. In all cases, the correlation remained with the same tendency. Despite its limitations, the selected vegetation model is the most accurate geographic information system we currently have for habitat description. A key element to be checked in the future is to include not only *Themeda* but also *K. scurra* host species in the vegetation model. Also, working with smaller scales could be appropriate especially when dispersal barriers are present as in the Windellama cemetery, or when encountering non‐native areas that nonetheless qualify as *K. scurra* habitats, such as at Goulburn and Wallendbeen. This modification would decrease the vegetation values of the first two and increase the value of the others.

The substantial genetic distances separating populations raise the issue of how to conserve diversity within the species. Clearly at this stage, genetic uniqueness of populations is not associated with a loss of genetic diversity as is the case of marsupials and some other invertebrates (Weeks et al., 2016). It is important to conserve current levels of diversity across the landscape, and the genomic data suggest that this can be achieved with relatively small areas. Increasing the number of fragments also helps protect against fires and other catastrophes that threaten Australia's insect species more generally (Sands, 2018), and provides nearby populations for future translocation efforts. The recreation of vegetation dominated by *Themeda* and a range of daisies is tractable if the high costs of seed can be overcome (Gibson‐Roy & Delpratt, 2015), so that the strategic creation of insurance populations of *K. scurra* is likely possible. Habitat corridors may have limited benefit for this species given that it can persist in small areas although its inability to fly means that any movement between nearby fragments will be rare.

Beyond their conservation merit, the ability to create populations may also permit studies of fundamental biological questions. Following on from Michael White's early work with the benefit of modern molecular tools, there are opportunities to further understand the evolutionary dynamics of *K. scurra* populations and reconsider some key evolutionary questions that were previously considered in this system. Early work by White argued that chromosomal rearrangements which could easily be scored in this grasshopper represented examples of heterozygote advantage and adaptive fitness interactions among chromosomal forms (White, 1957; White et al., 1963), which were interpreted as chromosomal forms being at different fitness peaks in an adaptive landscape (Lewontin & White, 1960). This was queried by others who argued for the importance of weak inbreeding (Allard & Wehrhahn, 1964) and changes in the selective advantage of different chromosomal arrangements across time (Colgan & Cheney, 1980) in accounting for patterns in these arrangements. By establishing populations with different combinations of chromosomal rearrangements from the same or different populations along climate gradients where the species occurs, and tracking changes in both the frequency of the rearrangements and their genomic content, it should be possible to gain insights into the extent to which rearrangements lock up adaptive genetic combinations, enhance or retard rates of evolutionary change, and change in fitness as a consequence of environmental variation. Such issues continue to be debated in the literature where *Drosophila* inversions in particular are regarded as important in climate adaptation (Kapun et al., 2016; Rane et al., 2015). Efforts to pursue these questions with *Keyacris scurra* would be greatly enhanced by developing an assembled and annotated genome of this and related morabine species.

## CONFLICT OF INTEREST

None declared.

## AUTHOR CONTRIBUTION

**Ary A. Hoffmann:** Conceptualization (lead); Funding acquisition (equal); Investigation (equal); Methodology (equal); Resources (lead); Supervision (lead); Writing‐original draft (lead). **Vanessa White:** Formal analysis (equal); Investigation (equal); Writing‐review & editing (supporting). **Moshe Jasper:** Formal analysis (equal). **Hiromi Yagui:** Investigation (equal); Methodology (equal); Writing‐review & editing (equal). **Steve J Sinclair:** Methodology (equal); Writing‐review & editing (equal). **Michael Kearney:** Funding acquisition (equal); Methodology (equal); Resources (equal); Supervision (equal); Writing‐review & editing (equal).

## Supporting information

Supplementary Material

## Data Availability

Aligned.bam sequence files for *Keyacris scurra* are available through https://www.ncbi.nlm.nih.gov/bioproject/702007.
