# Supplementary material for: An endangered flightless grasshopper with strong genetic structure maintains population genetic variation despite extensive habitat loss
Source: Ecol Evol. 2021 Apr 4;11(10):5364–80. doi: 10.1002/ece3.7428 (PMC8131777; doi:10.1002/ece3.7428)
Supplement: Supplementary file 1 — Supplementary Material [file ECE3-11-5364-s001.docx]

Supplementary. Description of unpublished Grassland spatial model

Source

Steve J. Sinclair and Matt D. White

Arthur Rylah Institute for Environmental Research, Victorian State Government Department of Environment, Land, Water and Planning. 123 Brown St, Heidelberg, Victoria, 3084.

Contact: Steve.sinclair@delwp.vic.gov.au

Introduction

The Victorian Government required a spatial model representing the distribution of Spiny Rice-flower (*Pimelea spinescens*). This species is largely restricted to grasslands dominated by Kangaroo Grass (*Themeda triandra*) that have never been cropped. A model was thus required that took into account cropping, which can be achieved by using satellite images as key inputs. A draft model was developed. It is still under development, and has not been fully tested nor published. Examination of the draft model shows that it predicts cover of many native grass species; beyond its intended targets of Kangaroo Grass and Spiny Rice flower (i.e. it over-predicts), but that this is a useful proxy for native grassy habitat in general. Catchment Management Authority staff have inspected this model (qualitatively) and found it to be more useful than existing maps in predicting the occurrence of diverse native grassy ecosystems. Given this, the draft model is being used in a limited capacity, while being further refined (in 2020-21). The draft model is used in this paper.

Methods

The model was ensembles of multi-target bagged random forests, implemented in the open-access platform CLUS (https://dtai.cs.kuleuven.be/clus) (Blockeel et al. 1999). The model predicted three targets:

- Grassland dominated by *Themeda*,
- Grasslands dominated by *Themeda* AND supporting *Pimelea*, and
- Not native.

The model was supplied with 61 spatial layers covering south Eastern Australia, to act as explanatory variables. These were of two kinds:

- The following indices derived from Landsat bands, each expressed as the median of multiple images across years for one of four seasons (Sep-Nov, Dec-Feb, Mar-May, Jun-Aug):
- Normalised Difference Vegetation Index (NDVI).
- Normalised Difference Moisture Index (NDMI).
- Normalised Difference Soil Index (NDSI).
- Normalised Burn Ratio (NBR).
- Enhanced Vegetation Index (EVI).
- Pre-existing layers created by the Victorian Government that represent the probabilities of land cover classes occurring at a pixel (woody vegetation, herbaceous wetland, shrublands).

The training data were extracted from known sites representing the three target classes:

- Grassland dominated by *Themeda*, compiled from disparate data sources (n = 865)
- Grasslands dominated by *Themeda* AND supporting *Pimelea*, taken from data collated by the Spiny Rice-flower recovery team (n = 1265).
- Not native; 53,815 random background points (assumed to be not native grassland) + 788 points selected to cover unusual places in the imagery (e.g. salt lakes, bare ground, buildings, etc).

All of the training sites were within Victoria. They are shown in Figure 1. Estimated areas around Keyacris collection sites are given in Table 1.

The model outputs are represented as three continuous values per pixel, one for each target. Because the targets are nested (*Themeda* + *Pimelea* is a subset of *Themeda*), the first split in the prediction is between *Themeda* and ‘not native’. The prediction for *Themeda* is used here.

Limitations

The model has the following key limitations, when used in the current context:

- It was trained only within Victoria, and extrapolated to New South Wales. There are not yet any data to verify model performance when spatially extrapolated.
- Its predictions are assumed to represent all native grassy systems (not just the *Themeda* dominated systems which were the target) on the basis of informal inspection of the model outputs only; not formal tests.
- It is known whether the prediction value for *Themeda* correlates with grass cover per se, or with the probability of *Themeda* dominated grassland being present at the site.


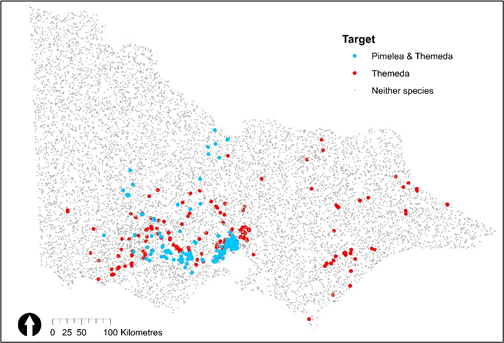


Figure 1. The three classes of training data.

Reference

BLOCKEEL, H., DŽEROSKI, S., & GRBOVIĆ, J. 1999. Simultaneous prediction of multiple chemical parameters of river water quality with TILDE. Pages 32–40 in JM Zytkow, Rauch J, editors. In European Conference on Principles of Data Mining and Knowledge Discovery. Springer, Berlin, Heidelberg.

Table 1. Grassland values used in the correlation analysis

| **Population** | **Buffer_25m** | **Buffer_50m** | **Buffer_100m** | **Buffer_250m** | **Buffer_500m** |
| --- | --- | --- | --- | --- | --- |
| Boorowa | 42 | 248 | 987 | 3330 | 10544 |
| Bungendore | 194 | 474 | 1954 | 10093 | 30911 |
| Bungonia | 13 | 202 | 930 | 7186 | 33934 |
| Burra | 225 | 625 | 2340 | 10117 | 28860 |
| Cooma | 121 | 401 | 1324 | 6742 | 24480 |
| Goulburn | 10 | 49 | 143 | 3173 | 24822 |
| Gundagai | 17 | 71 | 311 | 2350 | 8412 |
| Gundary | 288 | 810 | 2789 | 12101 | 40417 |
| Hall | 109 | 277 | 1334 | 10925 | 35234 |
| Kambah_Pool | 181 | 484 | 1960 | 7499 | 21372 |
| Lake_Omeo | 28 | 75 | 548 | 1732 | 5311 |
| Mulligan | 72 | 342 | 1345 | 7233 | 27251 |
| Omeo | 29 | 62 | 875 | 5666 | 23057 |
| SW_Omeo | 8 | 57 | 408 | 2938 | 14338 |
| Tarago | 48 | 127 | 482 | 3639 | 18740 |
| Wallenbeen | 7 | 21 | 105 | 1637 | 6336 |
| Windellama | 260 | 727 | 1836 | 8948 | 37023 |
